# The PTM switches of HBx: Master regulators of HBV infection and liver oncogenesis

**DOI:** 10.1016/j.virusres.2026.199781

**Published:** 2026-07-23

**Authors:** Zubing Ye, Meijuan Ma, Di Yu, Yibo Hu, Yan Liu, Jun Zhao, Zhanhai Su, Xiaohui Zhao

**Affiliations:** a Department of Pathogen Biology and Immunology, School of Medicine, Qinghai University, Qinghai, China; b Department of Orthopaedic Trauma, The Affiliated Hospital of Qinghai University, Qinghai, China; c Highland Cardio-Cerebrovascular Health Engineering Medicine Joint Research Center, Qinghai University, China; d Qinghai Institute of Technology, China

**Keywords:** HBx protein, Post-translational modifications, Hepatitis B virus, Viral infection, Hepatocellular carcinoma

## Abstract

The hepatitis B virus (HBV) X protein (HBx) functions as a versatile regulatory molecule that is indispensable for both efficient HBV replication and the pathogenesis of HBV-related hepatocellular carcinoma (HCC). Accumulating studies indicate that a broad spectrum of post-translational modifications (PTMs), including ubiquitination, phosphorylation, NEDDylation and ISGylation, critically influence HBx stability, intracellular trafficking, and its capacity to engage with diverse host factors. Collectively, these PTMs constitute a complex modulatory network that governs viral persistence and contributes to malignant transformation in the liver. In this review, we synthesize recent advances in deciphering the molecular principles that drive HBx PTMs and delineate their functional consequences during HBV infection and HCC development. We also summarize current understanding of how HBV manipulates host PTM systems to optimize its life cycle while promoting oncogenic processes. Furthermore, the review discusses emerging therapeutic opportunities centered on modulating HBx PTMs, with the aim of informing the design of next-generation antiviral and anti-tumor interventions.

## Highlights

• HBx post-translational modifications (PTMs) regulate HBV infection and hepatocarcinogenesis.
• Ubiquitination, phosphorylation, NEDDylation, and ISGylation control HBx stability and function.
• Targeting these PTMs via PROTACs, DUB or Pin1 inhibitors offers new therapeutic strategies.

## 1 Introduction

**Fig. 1:**
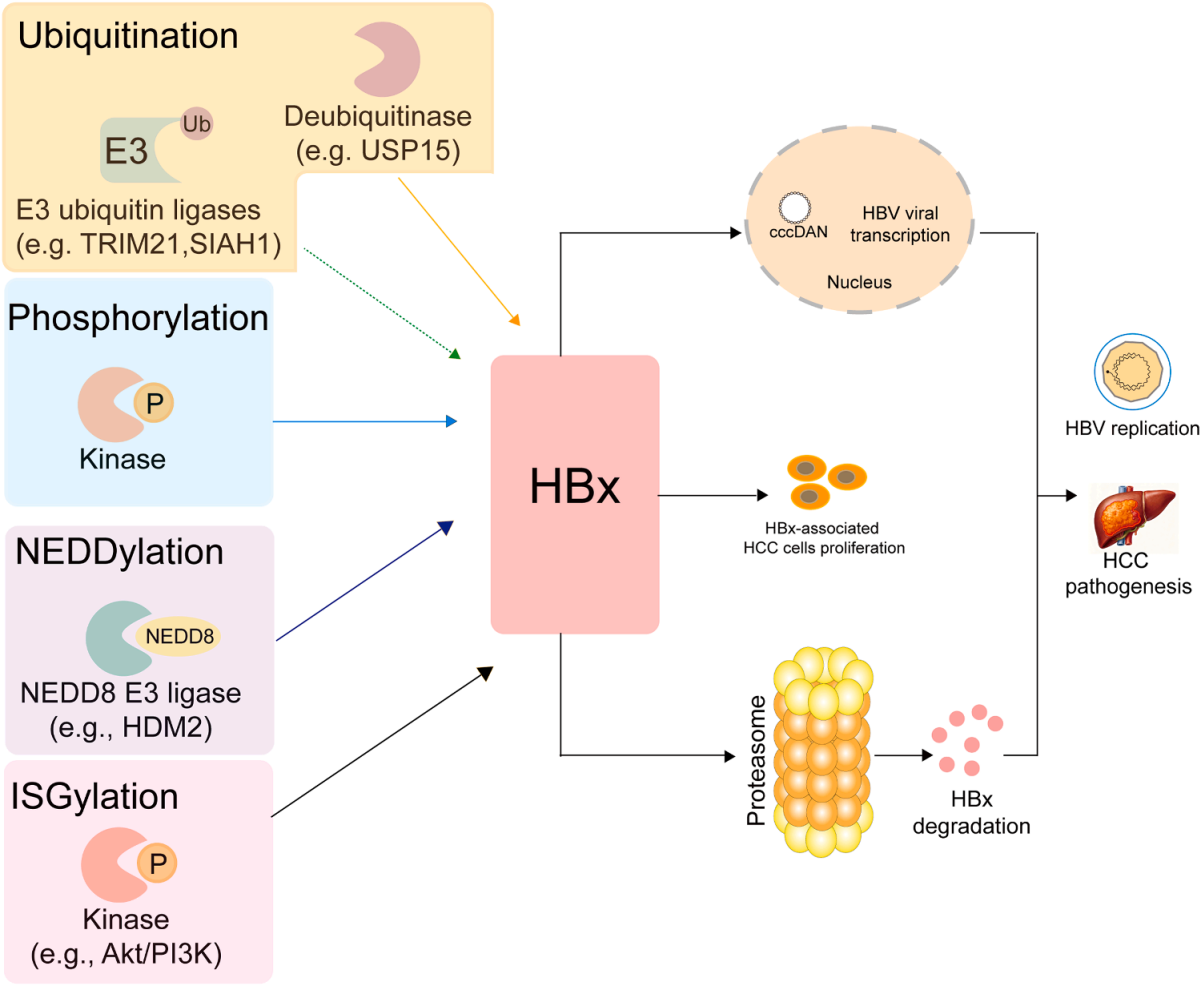
Schematic overview of post-translational modifications (PTMs) of HBx regulating HBx functions in HBV infection and hepatocarcinogenesis. This figure illustrates the four major PTMs discussed in this review: ubiquitination, phosphorylation, NEDDylation, and ISGylation, and their roles in modulating HBx stability, subcellular localization, and interactions with host factors. These modifications collectively influence HBV replication and contribute to the development of hepatocellular carcinoma (HCC). The central HBx protein is surrounded by representative modifying enzymes and the corresponding functional outcomes, including proteasomal degradation, transcriptional activation, and modulation of viral replication.

Hepatocellular carcinoma (HCC) ranks as the sixth most frequently diagnosed malignancy globally and remains the third leading cause of cancer‑associated mortality, with chronic HBV infection contributing to nearly half of all cases (Hwang et al., 2025). The HBV‑derived 17‑kDa HBx protein is indispensable for the viral life cycle and exerts profound oncogenic influence by participating in transcriptional control, modulating signaling pathways, and altering epigenetic states (Schollmeier et al., 2023). Although HBx does not possess intrinsic enzymatic activity, it functions as a versatile scaffold that engages a wide array of host factors to reprogram cellular machinery (Benhenda et al., 2009). Increasing evidence demonstrates that its interactions and biological activities are finely tuned by multiple post‑translational modifications (PTMs), which regulate HBx stability, functional output, and subcellular distribution in a dynamic manner (Kim et al., 2012; Shan et al., 2021; Liu et al., 2017). The extensive interplay among these PTMs forms an integrated regulatory network that facilitates persistent HBV infection while simultaneously promoting hepatocarcinogenesis. In this review, we examine four principal categories of HBx-related PTMs—ubiquitination, phosphorylation, NEDDylation and ISGylation—and summarize how these modifications shape HBx function during viral replication and liver tumorigenesis. We further emphasize their emerging potential as therapeutic intervention points for the treatment of HBV-associated diseases (Fig. 1).

## 2 Ubiquitination of HBx in HBV infection

### 2.1 The HBx-DDB1-SMC5/6 axis: a central framework for understanding HBx function

Before examining individual PTMs, it is essential to recall the central mechanism by which HBx regulates HBV transcription. HBx recruits the DDB1-containing CUL4 E3 ubiquitin ligase complex to target the SMC5/6 complex for K48-linked polyubiquitination and proteasomal degradation (Decorsiere et al., 2016; Murphy et al., 2016; Riviere et al., 2019). SMC5/6 acts as a restriction factor that binds to cccDNA and represses its transcription; thus, HBx-mediated degradation of SMC5/6 derepresses cccDNA transcription and promotes viral replication (Niu et al., 2017; Riviere et al., 2019). This ubiquitin-dependent degradation of a restriction factor is arguably the most well-defined function of HBx in the viral life cycle.

Given that ubiquitination lies at the heart of this regulatory axis, it is plausible that HBx PTMs–particularly those affecting HBx stability, DDB1 binding affinity, subcellular localization, or the efficiency of SMC5/6 recruitment–could profoundly influence cccDNA transcriptional output. However, as will become evident in the following sections, the majority of current studies on HBx PTMs have not directly examined their impact on the HBx-DDB1-SMC5/6 axis. Whether the ubiquitination, phosphorylation, NEDDylation, or ISGylation of HBx modulates SMC5/6 degradation and thereby affects cccDNA transcription–remains largely unexplored. This represents a critical gap in the field and an important direction for future research.

### 2.2 Ubiquitination of HBx in suppressing HBV infection

**Fig. 2:**
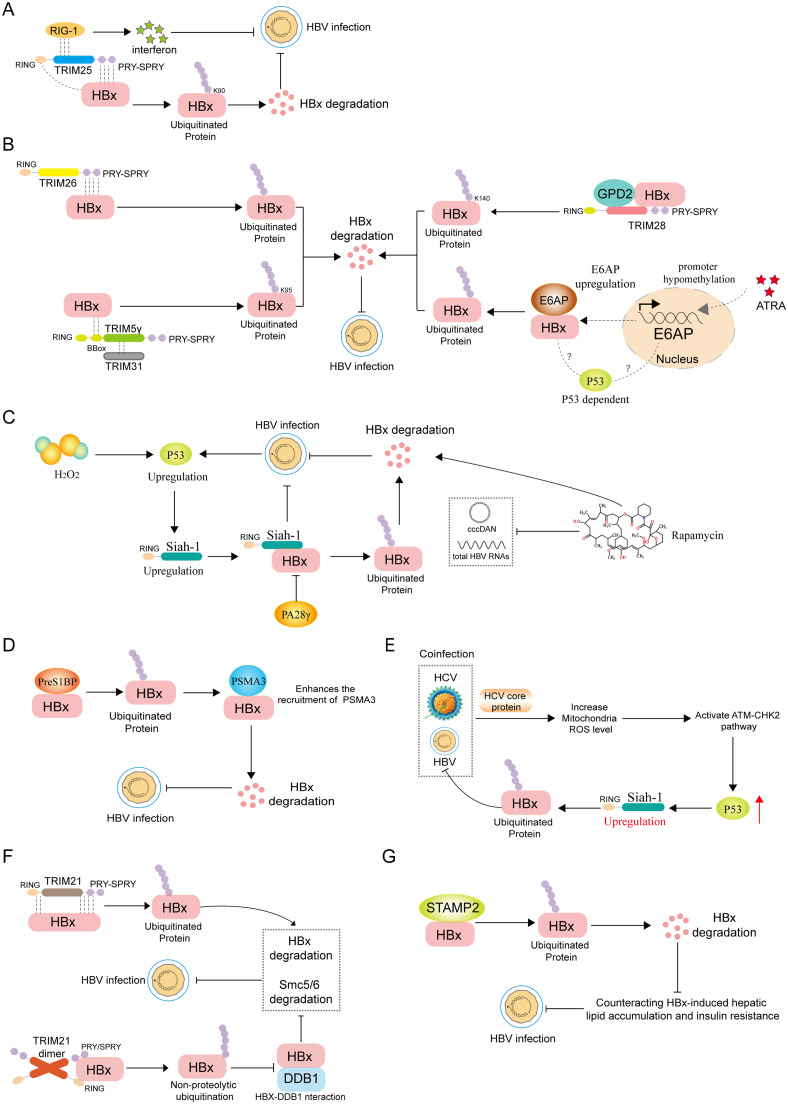
Ubiquitination of HBx in suppressing HBV replication. (A).TRIM25 associates with HBx and catalyzes ubiquitination at the K90 residue, promoting HBx degradation and restraining viral propagation. Enhanced TRIM25 expression also strengthens HBV pgRNA sensing through RIG-I interaction, amplifying interferon production. (B).All-trans retinoic acid (ATRA) promotes HBx ubiquitination via two pathways: strengthening E6AP-HBx interaction and activating Siah-1-mediated proteasomal degradation, both reducing HBx protein levels and suppressing HBV replication. (C).Rapamycin enhances HBx ubiquitination, leading to reduced cccDNA transcription and attenuated viral replication. (D).PreS1BP overexpression promotes recruitment of PSMA3 to HBx, accelerating its proteasomal degradation and suppressing HBV transcription and replication. (E).Hepatitis C virus (HCV) core protein downregulates HBx via Siah-1-mediated ubiquitination, providing mechanistic insight into HCV competitive dominance during HBV/HCV co-infection. (F).TRIM21 suppresses HBV replication via two distinct mechanisms: non-proteolytic HBx ubiquitination disrupting HBx-DDB1 binding or K48-linked HBx ubiquitination leading to HBx degradation. (G).STAMP2 interacts with HBx and promotes its ubiquitin-mediated degradation, simultaneously alleviating HBx-induced lipid accumulation and insulin resistance while repressing HBV gene expression.

The stability and regulatory mechanisms of the HBx protein play a critical role in HBV replication, and recent studies have gradually uncovered how various cellular factors and the ubiquitin–proteasome system finely regulate HBx. TRIM25 has also been shown to associate with HBx and catalyze ubiquitination at the K90 residue, promoting HBx degradation and consequently restraining viral propagation; in parallel, enhanced TRIM25 expression strengthens HBV pgRNA sensing through its interaction with RIG‑I, further amplifying interferon production (Song et al., 2023) (Fig. 2A). While these data point to a potential antiviral function of TRIM25, it should be borne in mind that the ubiquitination assays were performed in overexpression systems; whether endogenous TRIM25 similarly modifies HBx during natural infection remains unexplored. Additional evidence indicates that TRIM26 modulates viral replication, as its depletion elevates HBV levels whereas its overexpression exerts an inhibitory effect. Mechanistically, TRIM26 physically associates and co-localizes with HBx via the SPRY domain, facilitating ubiquitin-mediated degradation of the protein (Luo et al., 2022). These biochemical observations are instructive, yet they derive primarily from transfected cell systems and await confirmation in the context of authentic HBV infection. Interferon‑induced TRIM5γ has been reported to recruit TRIM31 to promote HBx turnover, thereby diminishing HBV replication (Tan et al., 2019). Notably, however, the evidence for this regulatory axis comes largely from overexpression studies, leaving open the question of whether it operates under physiological infection conditions. Liu et.al reported that overexpression of GPD2 likewise reduces viral replication in hydrodynamic injection mouse models, an effect attributed to GPD2‑dependent recruitment of the E3 ligase TRIM28 to enhance HBx degradation, independent of alterations in G3P metabolism (Liu et al., 2023a). It is worth noting that, although the hydrodynamic injection model provides in vivo evidence of HBV replication, it does not fully recapitulate the natural infection cycle, and the mechanistic dissection of HBx degradation was performed in overexpression systems. Han et.al revealed that All‑trans retinoic acid (ATRA) strengthens the interaction between E6AP and HBx, markedly increasing HBx ubiquitination and reducing its abundance, which results in repression of HBV replication (Han and Jang, 2024). Besides this pathway, ATRA additionally lowers HBx protein levels by activating Siah‑1–mediated proteasomal degradation, further restricting viral proliferation (Han and Jang, 2023) (Fig. 2B). Oxidative stress, frequently elevated in HBV‑infected hepatocytes, also exerts regulatory influence. Hydrogen peroxide exposure has been shown to reduce HBx levels through Siah‑1–dependent degradation, ultimately suppressing viral replication (Yoon et al., 2023). Rapamycin similarly inhibits HBV covalently closed circular DNA transcription by enhancing HBx ubiquitination, resulting in attenuated viral replication (Zhang et al., 2022) (Fig. 2C). A study revealed that PreS1BP overexpression markedly suppresses HBV transcription and replication by promoting the recruitment of PSMA3 to HBx, thereby accelerating its proteasomal degradation (Wang et al., 2024) (Fig. 2D). A study reported that the tumor suppressor p53 likewise exerts antiviral effects by decreasing HBx levels through E6AP‑mediated proteasomal degradation (Lim et al., 2022). Cross-viral interactions may also affect HBx stability. The hepatitis C virus core protein downregulates HBx via Siah-1–mediated ubiquitination, providing mechanistic insight into the competitive dominance of HCV during HBV/HCV co-infection (Lee et al., 2021) (Fig. 2E). Sheng et al. demonstrated that the PRYSPRY and RING domains of the TRIM21 dimer form a structural interface that accommodates HBx binding, and in overexpression and stable cell line systems, they found that TRIM21 induces non-proteolytic ubiquitination of HBx at residues T81/S104, which disrupts the HBx-DDB1 interaction and suppresses HBV replication in plasmid-based cccDNA reporter assays, without altering HBx protein stability (Sheng et al., 2024) (Fig. 2F). In a separate study, Song et al. reported that TRIM21 targets HBx for K48-linked polyubiquitination and proteasomal degradation in transfected hepatoma cells, thereby counteracting HBx-mediated Smc6 degradation and reducing viral replication (Song et al., 2021) (Fig. 2F). The discrepancy between these two studies regarding HBx stability warrants critical consideration. Song et al. reported proteasomal degradation of HBx, whereas Sheng et al. observed non-proteolytic ubiquitination that disrupts HBx-DDB1 interaction without affecting HBx protein levels. Nevertheless, both agree that TRIM21-mediated HBx ubiquitination suppresses HBV replication—either through HBx degradation or through impairing HBx-DDB1-mediated SMC5/6 degradation. However, both studies relied primarily on overexpression systems, and whether these mechanisms operate at endogenous HBx levels during authentic HBV infection remains to be determined. Furthermore, STAMP2 interacts with HBx and diminishes its stability through ubiquitin‑mediated degradation, simultaneously alleviating HBx‑induced lipid accumulation and insulin resistance and repressing HBV gene expression (Kim et al., 2012) (Fig. 2G).

### 2.3 Ubiquitination of HBx in promoting HBV infection

**Fig. 3:**
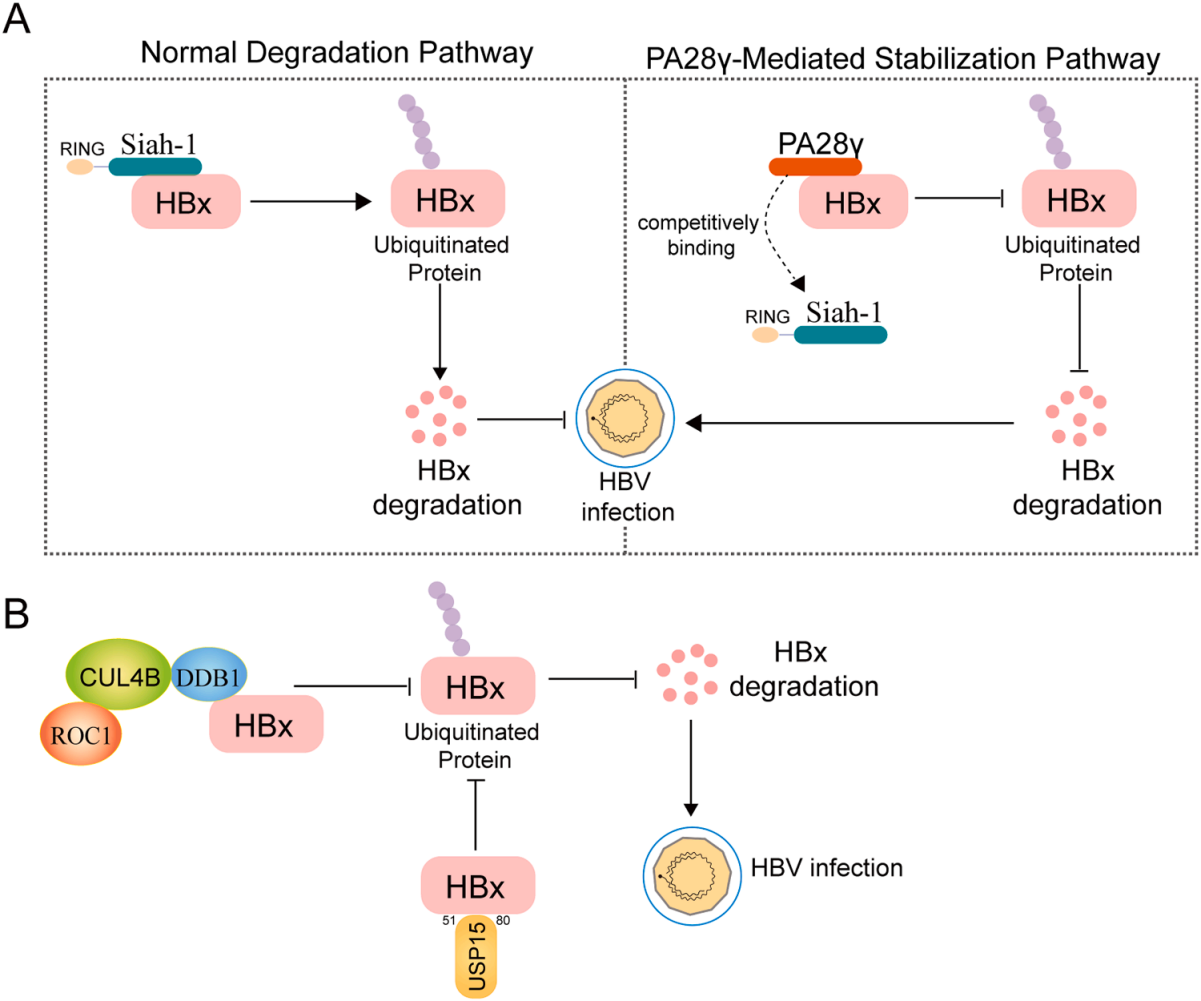
Ubiquitination-related mechanisms that promote HBV infection by stabilizing HBx. (A).PA28γ elevates HBx abundance and promotes HBV replication by competing with Siah-1 for HBx binding, thereby preventing Siah-1-driven proteasomal degradation. PA28γ acts as a molecular antagonist of Siah-1, competitively inhibiting Siah-1-HBx interaction, reducing HBx ubiquitination, and increasing HBx stability. (B).USP15 functions as a deubiquitinating enzyme (DUB) that removes ubiquitin chains from HBx, enhancing its stability and transactivation capacity, ultimately promoting HBV replication.

Ubiquitin‑mediated modulation of HBx has also been recognized as an active driver that enhances HBx function and thereby promotes HBV infection and replication. PA28γ elevates HBx abundance and promotes HBV replication by competing with Siah‑1 for HBx binding, thereby preventing Siah‑1–driven proteolysis (Han et al., 2021) (Fig. 3A). An important caveat is that the competition mechanism was delineated in transfected cells; its relevance in the setting of natural HBV infection has yet to be established. Shan et al. reported that CUL4B interacts with HBx and protects it from ubiquitination and proteasomal degradation in overexpression systems, thereby increasing HBx stability and promoting HBV replication in hydrodynamic injection mouse models. However, how CUL4B—an E3 ligase scaffold that typically promotes substrate ubiquitination—suppresses HBx ubiquitination remains mechanistically unclear. Notably, the same CUL4B E3 ligase machinery is recruited by the HBx-DDB1 complex to degrade SMC5/6, a central mechanism for cccDNA transcriptional activation (Decorsiere et al., 2016; Murphy et al., 2016). How CUL4B promotes SMC5/6 degradation while potentially stabilizing HBx represents an unresolved paradox that awaits validation in authentic infection models (Shan et al., 2021). Deubiquitinating enzymes also participate in HBx regulation. USP15 removes ubiquitin from HBx, enhancing its stability and transactivation capacity, ultimately promoting HBV replication (Su et al., 2017) (Fig. 3B). While this study identified USP15 as a positive regulator of HBx in transfected hepatoma cells, critical questions remain: does endogenous USP15 deubiquitinate HBx during natural infection, and does this modification influence cccDNA transcription through the SMC5/6 axis?

## 3 Ubiquitination of HBx in hepatocarcinogenesis

**Fig. 4:**
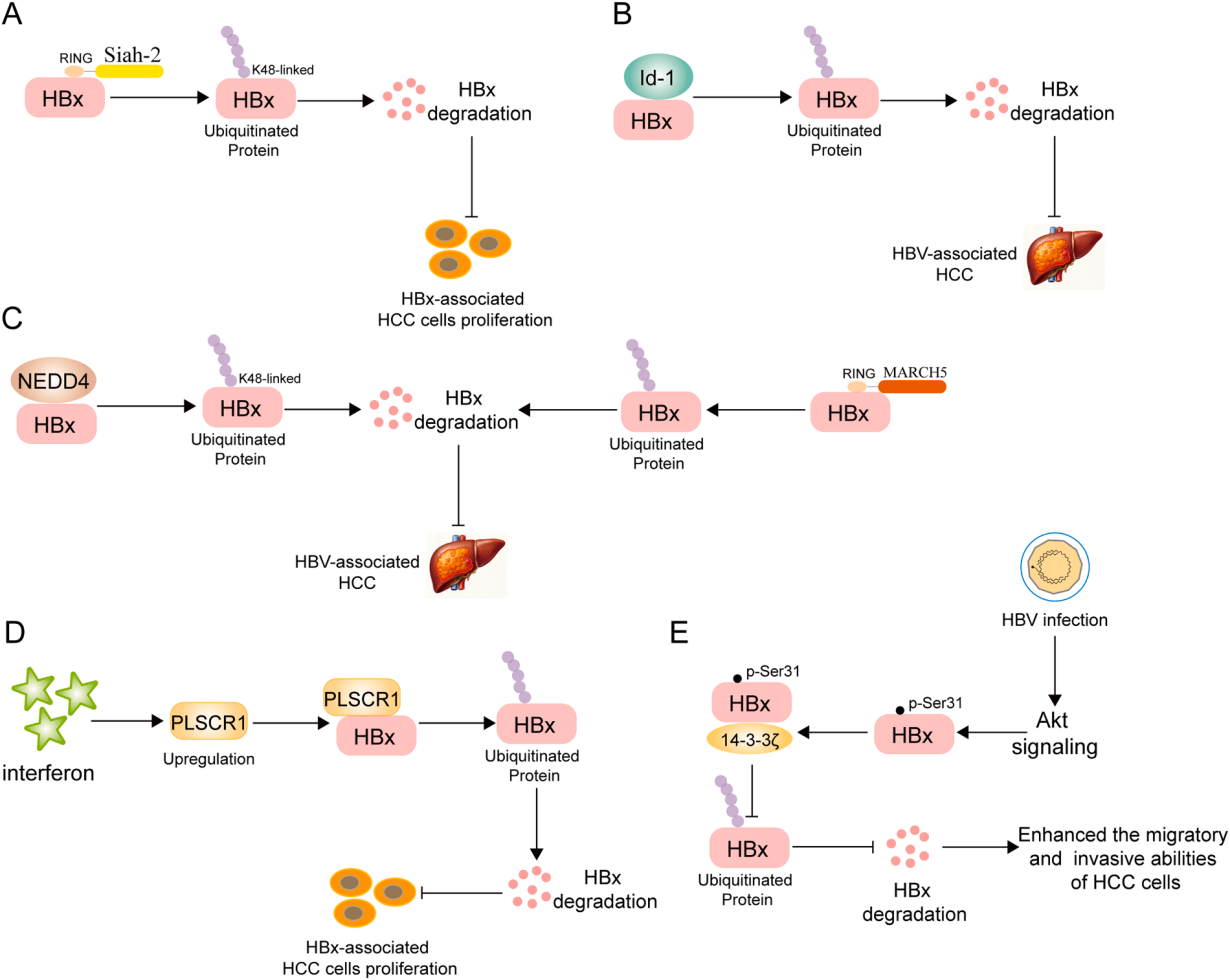
Ubiquitination of HBx in hepatocarcinogenesis. (A).SIAH2 (seven in absentia homologue 2) physically associates with HBx, promoting its K48-linked polyubiquitination and subsequent proteasomal elimination, thereby restraining HBx-driven proliferative activity in HCC cells. (B).Id-1, a helix-loop-helix (HLH) family protein, binds to HBx and enhances its ubiquitination in hepatocellular carcinoma models, contributing to the regulation of cell growth and tumor progression. (C).MARCH5, a mitochondrial E3 ubiquitin ligase, interacts with the mitochondria-localized pool of HBx and directs it toward degradation, thus attenuating hepatocarcinogenic processes. (D).PLSCR1, an interferon-inducible protein, accelerates HBx turnover through a ubiquitin- and proteasome-dependent mechanism, consequently suppressing HBx-mediated cell proliferation. (E).14–3–3ζ, a phosphopeptide-binding molecule frequently upregulated in HCC tissues, associates with HBx in a manner that diminishes its ubiquitination and stabilizes HBx expression in cancer cells and xenograft models, promoting tumor progression.

Beyond the factors previously identified as regulators of HBx stability, additional cellular proteins have been shown to modulate HBx turnover and consequently influence HBV‑related hepatocarcinogenesis. Recent findings indicate that SIAH2 (seven in absentia homologue 2) physically associates with HBx, promoting its K48-linked polyubiquitination and subsequent proteasomal elimination, thereby restraining HBx-driven proliferative activity in HCC cells (Hu et al., 2024) (Fig. 4A). Despite the appealing tumor-suppressive model proposed here, the experimental evidence is limited to HCC cell lines; whether SIAH2 similarly regulates HBx in the context of active HBV replication is unknown. Id‑1, a helix–loop–helix (HLH) family protein implicated in the control of cell growth, apoptosis, and senescence as well as in tumor progression, has also been shown to bind HBx and enhance its ubiquitination in hepatocellular carcinoma models (Ling et al., 2008) (Fig. 4B). Similarly, NEDD4 was reported to recognize the HBV X protein (HBx) and facilitate its K48‑dependent ubiquitin–proteasome degradation, ultimately exerting a tumor‑inhibitory effect in HBV‑related HCC (Wan et al., 2021). It should be emphasized that these observations were made in cancer cell lines and have not been extended to models of HBV infection; thus, the physiological significance of NEDD4-mediated HBx regulation remains uncertain. MARCH5, a mitochondrial E3 ubiquitin ligase, was found to interact with the mitochondria‑localized pool of HBx and direct it toward degradation, thus attenuating hepatocarcinogenic processes (Yoo et al., 2019) (Fig. 4C). One limitation of this study is its reliance on overexpression of both MARCH5 and HBx; whether endogenous mitochondrial HBx is subject to MARCH5-mediated degradation during HBV infection remains unclear. In addition, the interferon‑inducible protein PLSCR1 can accelerate HBx turnover through a ubiquitin‑ and proteasome‑reliant mechanism, consequently suppressing HBx‑mediated cell proliferation (Yuan et al., 2015) (Fig. 4D). As with many of the studies discussed here, these findings were generated in overexpression systems and await validation in the context of HBV replication. Conversely, 14‑3‑3ζ, a phosphopeptide‑binding molecule frequently upregulated in HCC tissues, was shown to associate with HBx in a manner that diminishes its ubiquitination and stabilizes HBx expression in cancer cells and xenograft models (Tang et al., 2018) (Fig. 4E). It is important to recognize that xenograft models, while useful for studying tumor growth, do not recapitulate the HBV-infected liver microenvironment; moreover, the mechanistic data were obtained primarily in cancer cell lines.

## 4 Phosphorylation of HBx in HBV infection and hepatocarcinogenesis

**Fig. 5:**
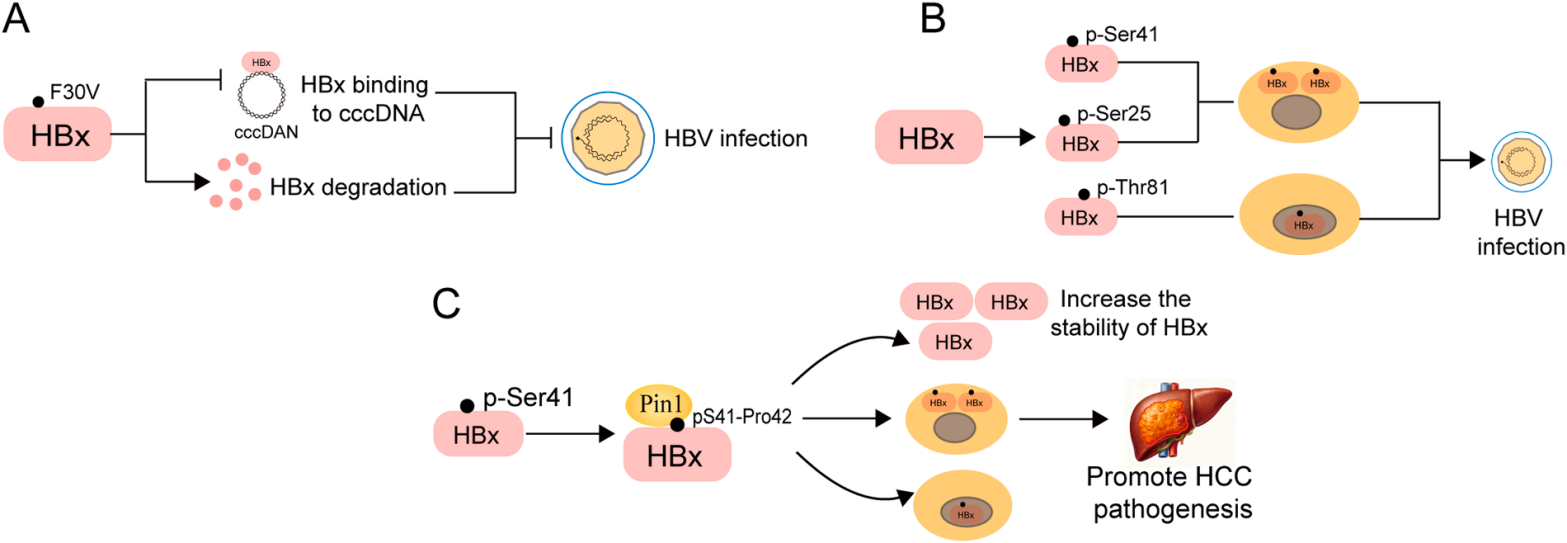
Phosphorylation of HBx in HBV infection and HCC. (A).The F30V substitution in HBx is associated with HBV-driven hepatocarcinogenesis in clinical cohorts and impairs viral replication by reducing HBx binding to cccDNA in transfection-based assays. (B).Phosphorylation at conserved residues Ser25, Ser41, and Thr81 may modulate the distinct functional roles of HBx arising from its differential subcellular distribution. (C).Pin1 is markedly overexpressed in HBV-associated HCC compared with non-HBV-related cases. Pin1 specifically engages the phosphorylated Ser41-Pro motif of HBx in a phosphorylation-dependent manner, thereby enhancing the transactivation potential of HBx and promoting HCC development.

Accumulating studies have revealed that phosphorylation‑dependent regulation of HBx plays a pivotal role in modulating its diverse biological activities and its contribution to HBV‑associated hepatocarcinogenesis. Salpini et al. identified the F30V substitution in HBx as a mutation associated with HBV-driven hepatocarcinogenesis in clinical cohorts. In transfection-based assays, this mutation was shown to diminish viral replication by impairing HBx binding to cccDNA while augmenting anti-apoptotic activity in vitro (Salpini et al., 2019) (Fig. 5A). The epidemiological link between F30V and HCC is compelling; however, the mechanistic connection between this mutation and HBx function in the context of authentic HBV infection—particularly regarding cccDNA transcription and the HBx-DDB1-SMC5/6 axis—remains to be elucidated. Lee et.al reported that the HBV X protein (HBx) itself has been linked to both pro‑ and anti‑apoptotic outcomes, and further evidence indicates that only the HBx isoform harboring an Akt phosphorylation site at Ser31 exerts anti‑apoptotic effects, whereas the form lacking this site displays pro‑apoptotic properties (Lee et al., 2012). Consistently, another investigation confirmed that Akt‑mediated phosphorylation of HBx at Ser31 is a key determinant of HBx‑driven hepatocarcinogenesis (Lee et al., 2016). In addition, using transfection-based mutagenesis studies in hepatoma cell lines, Prieto et al. proposed that phosphorylation at conserved residues Ser25, Ser41, and Thr81 may influence the subcellular distribution of HBx (Prieto et al., 2021) (Fig. 5B). While these observations offer a plausible model for how phosphorylation could regulate HBx localization, the clinical relevance of these phosphorylation events has not been established, nor have they been investigated in the setting of HBV infection. Pang et al. reported that Pin1 is markedly overexpressed in HBV-associated HCC compared with non-HBV-related cases in patient cohorts. Using biochemical and overexpression systems, the same study further demonstrated that Pin1 engages the phosphorylated Ser41-Pro motif of HBx and enhances its transactivation potential (Pang et al., 2007) (Fig. 5C). The clinical correlation between Pin1 overexpression and HBV-associated HCC is noteworthy; yet, the functional relevance of the Pin1-HBx interaction in the context of authentic HBV infection–particularly its impact on cccDNA transcription and the HBx-DDB1-SMC5/6 axis–has not been directly explored.

## 5 Other PTMs of HBx in HBV infection and hepatocarcinogenesis

**Fig. 6:**
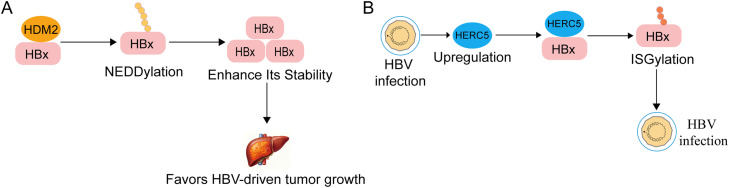
Other PTMs of HBx and their roles in HBV-associated HCC. (A).HDM2 facilitates HBx NEDDylation, thereby increasing HBx stability and enhancing its recruitment to chromatin. This ultimately promotes HBx-dependent transcriptional activity, cellular proliferation, and HBV-associated tumor development. (B).HERC5-driven ISGylation of HBx supports HBV replication and contributes to resistance against IFN-α-mediated antiviral responses.

Beyond phosphorylation, additional post‑translational modifications of HBx, particularly ubiquitin and ubiquitin‑like conjugations, have emerged as critical regulators of its stability and functional activities in HBV‑associated hepatocarcinogenesis. Ubiquitin and ubiquitin‑like modifiers, including NEDD8 (neural precursor cell expressed developmentally downregulated gene 8) and SUMO, can influence a substrate protein’s stability, intracellular distribution, and interaction networks. One study demonstrated that the E3 ligase HDM2 facilitates HBx NEDDylation, thereby increasing HBx stability and enhancing its recruitment to chromatin, ultimately promoting HBx‑dependent transcriptional activity, cellular proliferation, and HBV‑associated tumor development (Liu et al., 2017) (Fig. 6A). Of note, Liu et al. also found a positive correlation between HDM2 and HBx protein levels in 160 HCC specimens–a valuable clinical observation. Nevertheless, the mechanistic demonstrations of NEDDylation relied on overexpression systems, and whether HDM2 modifies endogenous HBx in the setting of authentic HBV infection–and whether this modification affects SMC5/6 degradation–has not been addressed. In addition, HERC5‑driven ISGylation of HBx has been shown to support HBV replication and contribute to resistance against IFN‑α‑mediated antiviral responses (Bawono et al., 2021) (Fig. 6B). It should be noted that the Hep38.7-Tet system, while supporting HBV replication, does not fully recapitulate the natural infection cycle (e.g., viral entry and cccDNA formation). Thus, whether HBx ISGylation occurs at endogenous HBx levels during authentic infection and whether it contributes to cccDNA transcription via the SMC5/6 axis remain open questions. Although acetylation of HBx has been detected when the protein is expressed in insect cells, the functional relevance of this modification to HBx‑related activities remains unclear (Bouchard and Schneider, 2004).

## 6 Therapeutic targeting of HBx

The essential functions of HBx in both the HBV replication cycle and hepatocarcinogenesis, together with its persistent expression in infected hepatocytes despite nucleos(t)ide analog treatment, underscore HBx as a compelling yet difficult therapeutic target (Revill et al., 2019). Unlike classical enzymatic proteins, HBx operates as a pleiotropic regulator without a defined catalytic pocket suitable for traditional small‑molecule inhibition (Slagle and Bouchard, 2016). Nevertheless, the complex array of PTMs that dictate HBx stability, intracellular trafficking, and functional activities creates exploitable therapeutic vulnerabilities. Increasing attention has therefore shifted toward strategies that modulate this “PTM code” to promote HBx degradation, interfere with its interaction partners, and mitigate its oncogenic potential. Such therapeutic concepts generally fall into several interrelated categories (Kumar, 2025, Slagle and Bouchard, 2018).

### 6.1 Targeting the ubiquitin-proteasome system to destabilize HBx

Targeting the ubiquitin–proteasome pathway to promote HBx destabilization has emerged as an attractive therapeutic concept, as it directly interferes with the finely regulated interplay between HBx ubiquitination and deubiquitination (Huang and Dixit, 2016). Strategies designed to accelerate HBx turnover primarily aim to enhance its ubiquitin tagging and subsequent proteasomal elimination. Two major approaches have been proposed. The first involves the development of small‑molecule inhibitors against HBx‑stabilizing deubiquitinating enzymes (DUBs), such as USP15; suppressing these DUBs biases HBx toward polyubiquitination, driving its rapid degradation and thereby impairing HBV replication and HBx‑dependent survival pathways (Su et al., 2017). The second relies on the application of Proteolysis‑Targeting Chimera (PROTAC) technology, which is particularly well suited for eliminating non‑enzymatic regulatory proteins like HBx (Ottis and Crews, 2017; Zhong et al., 2024). A HBx‑specific PROTAC comprises a bifunctional molecule that anchors HBx on one end while simultaneously recruiting an E3 ubiquitin ligase on the other, enabling catalytic ubiquitination and degradation of HBx (Montrose and Krissansen, 2014). Because this modality eradicates the entire protein instead of suppressing a single activity, it offers significant therapeutic potential. Nonetheless, both DUB‑targeting agents and PROTACs face a critical challenge: achieving precise selectivity, as DUBs and E3 ligases play indispensable roles in numerous cellular pathways (Komander et al., 2009; Liu et al., 2023b). Thus, the development of highly selective compounds with minimal off‑target effects is essential for advancing this therapeutic strategy.

### 6.2 Inhibiting HBx-Mediated signaling by targeting regulatory phosphorylation

The phosphorylation profile of HBx, particularly at Ser/Thr‑Pro motifs, plays a decisive role in regulating its association with critical signaling mediators such as Pin1. Because the prolyl isomerase Pin1 is essential for maintaining HBx stability and augmenting its transactivation capacity after phosphorylation, Pin1 has emerged as an attractive therapeutic target (Pang et al., 2007). A number of Pin1 inhibitors have been developed and demonstrated promising activity in preclinical cancer models (Guo et al., 2020; Cheng and Tse, 2019). Applying these inhibitors to HBV‑related HCC offers dual benefits: attenuation of HBx function and disruption of a pivotal hub within oncogenic signaling networks. Pharmacologic suppression of Pin1 would prevent phosphorylated HBx from undergoing the conformational change necessary for its full functional activation, thereby promoting its inactivation or subsequent degradation (Liao et al., 2017). Although less specific, an additional therapeutic concept involves identifying the kinases responsible for oncogenic phosphorylation events on HBx—such as those occurring at Ser‑28, Ser‑31, and Ser‑41, which could potentially enable the repurposing or development of kinase‑targeted agents. However, this strategy is complicated by extensive kinase redundancy and the high likelihood of broad off‑target effects (Shyam Sunder et al., 2023).

### 6.3 Challenges and caveats for PTM-targeted therapeutic strategies

Despite the conceptual appeal of targeting HBx PTMs, several significant challenges warrant consideration before these strategies can be realistically pursued for clinical development. First, HBx is expressed at very low levels during natural HBV infection, and it remains unclear whether PTM-directed approaches can achieve sufficiently efficient and selective HBx depletion to impact chronic infection, particularly given the low abundance of HBx derived from the cccDNA minichromosome (Kornyeyev et al., 2019). Second, the DUBs and E3 ligases that regulate HBx—including USP15, CUL4B, and various TRIM proteins—have pleiotropic functions in host cell homeostasis. Systemic inhibition of these enzymes carries a high risk of off-target toxicity (Harrigan et al., 2018; Deng et al., 2020). Achieving selective targeting of the HBx-associated pool of these enzymes without affecting their normal physiological functions represents a major pharmacological challenge. Third, the relevance of these PTMs in physiologically relevant infection models–such as primary human hepatocytes infected with cell culture-derived HBV or in vivo HBV replication models–has not been systematically established. Most mechanistic insights to date come from overexpression or transfection systems, and it is unclear whether the same PTMs occur at endogenous HBx levels during natural infection (Bao et al., 2024). Fourth, HBV infection persists chronically with continuous production of viral antigens and progeny virions. Even if HBx degradation could be achieved, the accumulated damage from chronic infection and the multifactorial nature of hepatocarcinogenesis suggest that HBx-targeting alone may not be sufficient to reverse established HCC or achieve a functional cure (Tsounis et al., 2021; Fanning et al., 2019).

Thus, while PTM-targeting strategies represent an innovative and conceptually attractive direction, their translational potential remains hypothetical at this stage. Substantial preclinical validation in authentic infection models, coupled with careful evaluation of selectivity and safety, will be required to determine whether these approaches can be developed into clinically meaningful interventions.

### 6.4 Synergistic strategies and future directions

Targeting the post-translational modifications of HBx alone is unlikely to produce a curative effect; rather, such approaches are more plausibly integrated into comprehensive, multipronged treatment regimens. Combination with Existing Antivirals: Strategies that promote HBx degradation or diminish its functional activity may partially reconstitute intrinsic antiviral defenses and attenuate cccDNA transcription, thereby improving the probability of attaining a functional cure when co-administered with polymerase inhibitors (Li et al., 2024). Combination with Immunotherapies: By mitigating HBx-driven immunosuppression and normalizing antigen-presentation processes, HBx-focused interventions have the potential to potentiate the therapeutic performance of immune checkpoint inhibitors in the management of HBV-associated HCC (Levrero and Zucman-Rossi, 2016; Chen et al., 2020).

## 7 Conclusion

In summary, the once‑enigmatic spectrum of HBx post‑translational modifications has evolved into a clearer framework that can guide therapeutic development. By selectively modulating the enzymatic machinery that write (kinases, acetyltransferases), erase (phosphatases, deacetylases, DUBs), or read (such as Pin1) the PTM signatures on HBx, it becomes feasible to destabilize the protein and disrupt its wide‑ranging biological activities with greater precision. Notably, HBx is not merely a recipient of multiple PTMs but also an active regulator of the modification patterns of numerous host proteins, thereby extending its impact across critical cellular signaling networks. This capacity to reshape the host PTM environment underscores HBx as a central orchestrator of the intracellular milieu and reinforces its value as a therapeutic target. Although substantial obstacles concerning drug specificity and efficient delivery persist, advancing strategies that interfere with the PTMs of HBx—and with the extensive PTM circuitry it modulates within host cells—marks a conceptual transition from inhibiting viral enzymes to intervening in the core regulatory systems exploited by this multifaceted viral protein to sustain chronic infection and promote oncogenesis.

## CRediT authorship contribution statement

**Zubing Ye:** Writing – original draft, Software. **Meijuan Ma:** Validation, Resources, Investigation. **Di Yu:** Writing – review & editing, Validation. **Yibo Hu:** Writing – review & editing, Writing – original draft. **Yan Liu:** Software, Resources. **Jun Zhao:** Software, Formal analysis. **Zhanhai Su:** Writing – review & editing, Supervision, Resources. **Xiaohui Zhao:** Writing – review & editing, Writing – original draft, Visualization, Supervision, Investigation.

## Declaration of competing interest

The authors declare that they have no known competing financial interests or personal relationships that could have appeared to influence the work reported in this paper. Authors: Zubing Ye, Meijuan Ma, Di Yu, Yibo Hu, Yan Liu, Jun Zhao, Zhanhai Su, Xiaohui Zhao.

## Data Availability

No data was used for the research described in the article.
